# Effect of 2020 containment strategies on trauma workflow in *Ile-de-France* region: another benefit of lockdown?

**DOI:** 10.1186/s13049-021-00918-x

**Published:** 2021-09-14

**Authors:** Jean-Denis Moyer, Paer-Selim Abback, Sophie Hamada, Thibault Martinez, Marie Werner, Arthur James, the Traumabase®Group

**Affiliations:** 1 Department of Anesthesiology and Critical Care, Beaujon Hospital, DMU Parabol, AP-HP.Nord, 100 boulevard du General Leclerc, F92110 Paris, Clichy France; 2 Department of Anesthesiology and Critical Care, Hôpital Européen Georges Pompidou, AP-HP, Paris, France; 3 Intensive Care Unit, Percy Military Teaching Hospital, 101 Avenue Henri Barbusse 92140, Clamart, Val de Grace Academy, Place Alphonse Laveran, 75005 Paris, France; 4 Department of Anesthesiology and Critical Care, AP-HP, Bicêtre Hôpitaux Universitaires Paris-Sud, Université Paris Saclay, Le Kremlin Bicêtre, France; 5 GRC 29, AP-HP, DMU DREAM, Department of Anaesthesiology and critical care, Pitié-Salpêtrière Hospital, Sorbonne University, Paris, France

**Keywords:** COVID-19, Trauma, France, Traumacenter, ICU capacity

## Abstract

**Background:**

During the SARS-CoV-2 pandemic, the French Government imposed various containment strategies, such as severe lockdown (SL) or moderate lockdown (ML). The aim of this study was to evaluate the effect of both strategies on severe trauma admissions and ICU capacity in *Ile-de-France* region (Paris Area).

**Main text:**

We conducted a multicenter cohort-based observational study from 1^st^January 2017 to 31th December 2020, including all consecutive trauma patients admitted to the trauma centers of *Ile-de-France* region participating in the national registry (Traumabase®). Two periods were defined, the “non-pandemic period” (NPP) from 2017 to 2019, and the “pandemic period” (PP) concerning those admitted in 2020. The number of ICU beds released during 2020 pandemic period (overall period, SL and ML) was estimated by multiplying difference in trauma admissions by the median length of stay during the same week of pandemic period (ICU day-beds in 2020).

A 15% yearly reduction of trauma patients was observed during the PP, associated with the release of 6422 ICU day-beds in 2020. During SL and ML, the observed decrease in trauma admission was respectively 49 and 39% compared with similar dates of the NPP. The number of beds released was 1531 days-beds in SL and 679 day-beds in ML. Those reductions respectively accounted for 4.5 and 6.0% of the overall ICU admission for COVID-19 in *Ile-de-France*.

**Conclusion:**

The lockdown strategies during pandemic resulted in a reduction of severe trauma admissions. In addition to the social distancing effect, lockdown strategies freed up an important number of ICU beds in trauma centers, available for severe COVID-19 patients.

## Background

Because of the first and the second waves of SARS-CoV-2 pandemic **in 2020**, a large number of patients were admitted to French intensive care units (ICU) [1, 2]. As a consequence of this massive influx of patients, ICU capacity had to be increased considerably. In order to absorb the SARS-CoV-2 burden and to limit the ICU overflow, the French government, as well as many others in the world, imposed strict containment strategies during the spring and fall waves. The Covitrauma study recently published in this journal as confirmed that the first lockdown reduced severe trauma admissions by 50% [3], indicating an indirect benefit of the lockdown by reducing non SARS-CoV-2 ICU emergency admissions. The aim of this commentary was to complete our first study through the evaluation of the effect of containment strategies on severe trauma admissions and ICU capacity in *Ile-de-France* region (Paris Area).

## Main text

We conducted a multicenter cohort-based observational study from 1^st^January 2017 to 31th December 2020. During each of these 4 years, all consecutive trauma admitted to the trauma centers of *Ile-de-France* region (12.2 million of inhabitants) participating in the national registry (Traumabase®) were included (5 teaching hospitals and one military teaching hospital). Patients were categorized in two groups according to their year of admission, allocating those admitted within years 2017, 2018 and 2019 to the “non-pandemic period” group and those admitted in 2020 to the “pandemic period” group. Each variable result over the non-pandemic period were summarized using its **median** (and interquartile) or number (and relative percentage).

During the pandemic period, two types of lockdown strategies were applied in *Ile de France* area:

- Severe lockdown (SL) from week 12 to week 19 **in 2020**: leaving home was only allowed for essential purchases, medical care, exercise (1-h session per day), and work journeys if work from home was impossible.
- Moderate lockdown (ML) from week 45 to week 51 **in 2020**: leaving home was only allowed for essential purchases, medical care, exercise (1-h session per day), and work journeys (work in factories and public works were allowed). Kindergarten, schools and high schools were opened.

The number of ICU beds released during 2020 pandemic period (overall period, SL and ML) was estimated by multiplying difference in trauma admissions (difference between median number of trauma admissions each week during the pandemic and the non-pandemic period) by the median length of stay during the same week of pandemic period (ICU day-beds in 2020).

Data for SARS-CoV-2 patients admitted in ICU in *Ile-de-France* region are available on open access data [4].

**Fig. 1:**
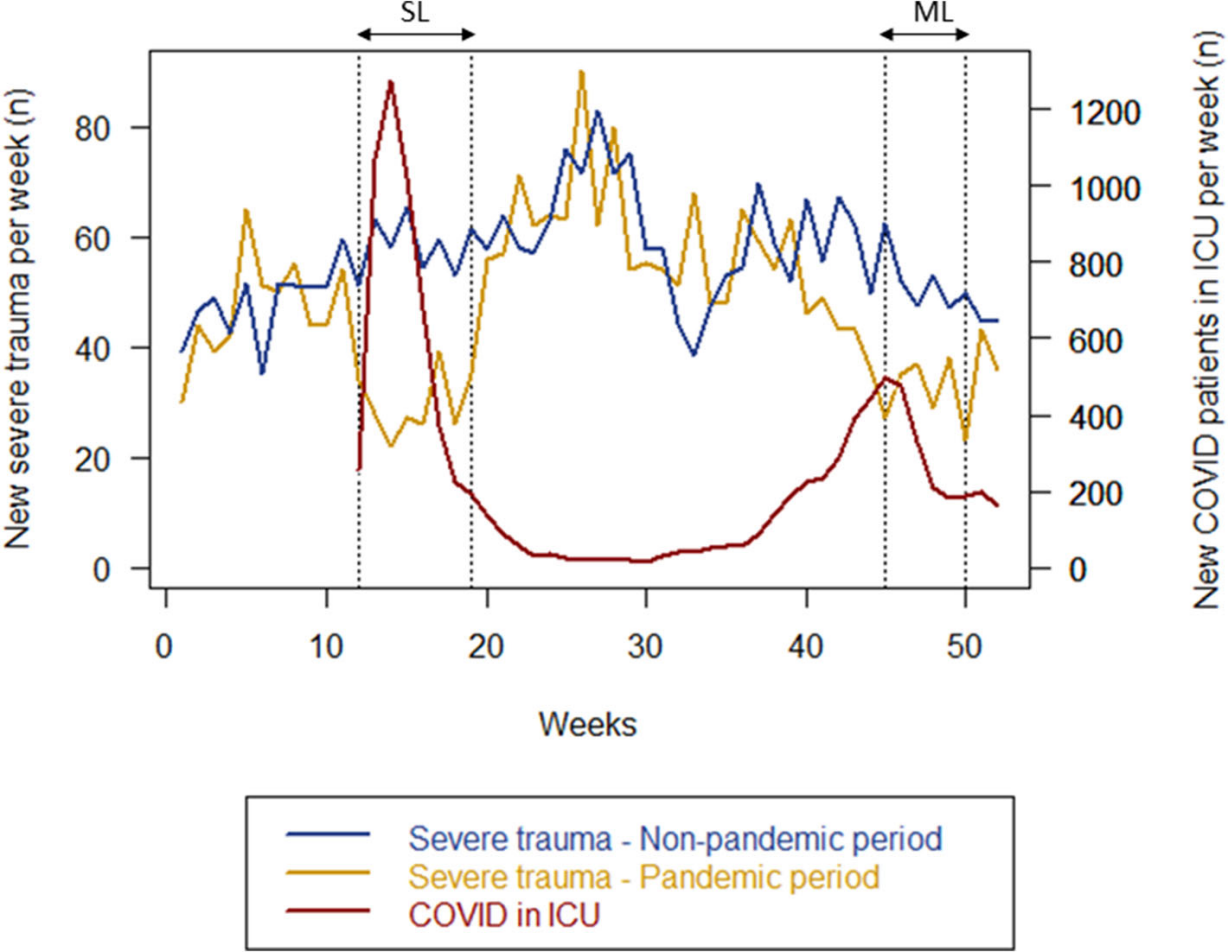
Weekly number of patients admitted to Trauma center and to *Ile-de-France* Intensive Care Unit for SARS-CoV2 infection in pandemic period (2020), compared with patients admitted to Trauma center during non-pandemic period (average of 2017–2019). SL: severe lockdown, ML: moderate lockdown

Over the 4 year-period, 11,230 trauma patients were admitted in the participating trauma centers, 8749 during the non-pandemic period (average 2916 per year) and 2481 during the pandemic period, representing a 15% yearly reduction during the pandemic period (Table 1 and Fig. 1). This observed 15% reduction between both periods was associated with the release of 6422 ICU day-beds in 2020. During SL and ML, the observed decrease in trauma admission was respectively 49 and 39% compared with similar dates of the non-pandemic period (Fig. 1 and Table 1). During the 8 weeks of SL and the 6 weeks of ML, the number of beds released by the reduction of the trauma activity was 1531 days-beds and 679 day-beds respectively. Those reductions of trauma admission respectively accounted for 4.5 and 6.0% of the overall ICU admission required for COVID-19 over the same period of time in *Ile-de-France*.

**Table 1:** Epidemiology of patients admitted to Trauma centers during pandemic period (2020) and non-pandemic period (2017–2019)

	Period					
	Overall year		Severe Lockdown		Moderate lockdown	
Variable	Non-pandemic	Pandemic	Non-pandemic	Pandemic (SL)	Non-pandemic	Pandemic (ML)
Number of admitted patients	2916^b^	2481	466^b^	237	312^b^	189
Age (Years)	36 [25, 52]	34 [23, 50] ^a^	37 [25, 53]	35 [26, 49]	36 [25, 54]	33 [23, 48] ^a^
Gender (Men)	2279^b^ (78.5)	2002 (82.2) ^a^	364^b^ (78.2)	196 (83.1)	235^b^ (76.1)	147 (78.2)
Mechanism of injury distribution						
Penetrating trauma	364^b^ (12.5)	387 (15.6) ^a^	55^b^ (11.8)	36 (15.2) ^a^	39^b^ (12.4)	34 (18.0) ^a^
Road traffic accident	1566^b^ (53.7)	1452 (50.5) ^a^	249^b^ (53.3)	93 (39.9) ^a^	170^b^ (54.7)	91 (48.1) ^a^
Fall from height	606^b^ (20.8)	546 (22.0) ^a^	97b (20.9)	86 (36.6) ^a^	55^b^ (17.8)	52 (27.5) ^a^
Other	381^b^ (13.1)	296 (11.9) ^a^	64^b^ (13.7)	12 (9.3) ^a^	47^b^ (15.2)	12 (6.3) ^a^
ISS	11 [5, 21]	11 [5, 20]	11 [5, 22]	12 [5, 20]	12 [5, 22]	13 [8, 22]
SAPS 2	20 [13, 34]	20 [13, 31]	21 [13, 34]	21 [13, 30]	21 [13, 36]	20 [13, 32]
Hemorrhagic shock	176^b^ (6.1)	141 (5.8)	33^b^ (7.1)	11 (4.8)	19^b^ (6.2)	11 (5.8)
Traumatic brain injury	707^b^ (24.4)	516 (21.7) ^a^	108^b^ (23.4)	44 (19.4)	80^b^ (26.2)	30 (16.5)
Surgery in the first 24 h	1452^b^ (49.8)	1226 (49.4)	240^b^ (51.6)	127 (53.6)	140^b^ (45.5)	96 (50.8)
In-hospital mortality	832^b^ (9.6)	171 (7.5) ^a^	133^b^ (9.6)	10 (4.6)	101^b^ (10.9)	14 (8.1)
ICU Length of stay (days)	2 [2, 6]	2 [2, 5]	2 [2, 6]	2 [2, 5]	2 [2, 7]	2 [2, 6]
Predicted mortality (TRISS, mean, SD) (%)	8.5 (19.1)	7.4 (17.9)	8.4 (19.3)	7.7 (19.2)	9.2 (19.8)	9.1 (20.2)
Non-pandemic period: 2017–2018-2019. Pandemic period: 2020						

^**a**^**highlight differences with*p*-value ≤ 0.01 between the pandemic and the non-pandemic period**

^**b**^**For the non-pandemic period, we divided number of categorial variables by 3 in order make a comparison possible**

**Data are n (%) or median [Q1, Q3]**

***ICU*Intensive Care Unit,*ISS*Injury Severity Score,*SAPS 2*Simplified Acute Physiology Score,*TRISS*Trauma Related Injury Severity Score**

***SL*severe lockdown;*ML*moderate lockdown**

The characteristics of the population during pandemic and non-pandemic period are described Table 1. Trauma severity assessed by Injury Severity Score did not differ during both periods (11 [5, 21] vs 10 [5, 21] *p* = 0.68). Mechanisms of injury differed markedly between both period: road traffic accidents decreased during both lockdown whereas proportion of penetrating trauma and fall from height significantly increased (but not the absolute numbers of event) (Table 1).

These results highlight how trauma epidemiology in Paris Area has been impacted during the pandemic period and especially during the SL and the ML. The national lockdowns imposed by authorities to control the pandemic reduced severe trauma incidence. This study suggests that lockdown benefice on SARS-CoV-2 pandemic management is not only related to the prospect of controlling virus circulation. The reduction of trauma center workflow during lockdown freed up an important number of ICU beds for critical care patients and this effect seems to be more pronounced with a severe lockdown rather than a moderate one. This “collateral effect” of lockdown partly protected our health care system when facing the overwhelming and sudden surge in the number of patients in need of urgent treatment, it allowed to reallocate these free ICU beds to SARS-CoV-2 patients and probably contributed to maintain a part of scheduled major surgical activity such as cancer, cardio-vascular surgery or transplantation. This important observation might help public health stakeholders when preparing for potential future pandemic waves.

Furthermore, during this period, trauma centers did not restrict their admission policy, as indicated by the similar median ISS across the study periods. The proportion of patients operated within the first 24 h remained identical suggesting timely access to complex trauma surgery was not impeded. The trauma system analyzed in this study provides adequate and appropriate care equivalent to non-pandemic period, while participating significantly to the 2020 COVID-19 surge.

Study limitations first include a lack of specific data on SARS-CoV-2 admission in Trauma centers. However, the official occupation rate of ICU in Paris area during pandemic waves was over 100% during SL and ML compared with ICU standard capacity. Secondly, this study only represents urban territory and should not be transposed in rural territories. Thirdly, data for non-trauma centers are not available; then the effect of lockdown on ICU beds release in non-trauma center could not be analyzed.

## Conclusion

The lockdown strategies during pandemic resulted in a reduction of severe trauma admissions. This reduction did not appear to be driven by modification of severe trauma admission policy or quality of care, although surge capacity may have impacted both. In addition to the social distancing effect, lockdown strategies freed up an important number of ICU beds in trauma centers, available for severe COVID-19 patients.

## Data Availability

The data that support the findings of this study are available from the Traumabase® group but restrictions apply to the availability of these data, which were used under license for the current study, and so are not publicly available. All data are however available from the authors upon reasonable request and with permission of the Traumabase® group. COVID-19 ICU related data are freely available (https://www.data.gouv.fr/fr/datasets/r/41b9bd2a-b5b6-4271-8878-e45a8902ef00).

## Abbreviations

ICU: Intensive care unit
SL: Severe lockdown
ML: Moderate lockdown
